# Acute onset psychiatric diseases after SARS-CoV-2 virus infection among pediatric patients

**DOI:** 10.3389/fneur.2024.1445903

**Published:** 2024-10-09

**Authors:** Lu Yang, Jun Li, Dongqing Zhang

**Affiliations:** Pediatric Department, Qilu Hospital, Shandong University, Jinan, China

**Keywords:** child, adolescent, psychiatric symptoms, COVID 19, SARS-CoV-2

## Abstract

**Background:**

Psychiatric symptoms directly associated with SARS-CoV-2 virus infection have been reported sporadically in children. More cases of new-onset psychosis without severe cardinal symptoms, altered consciousness level, and psychogenic drug usage would offer compelling grounds for the association between the virus infection and psychosis.

**Methods:**

We collected the clinical data of pediatric patients with new onset psychiatric symptoms after the SARS-CoV-2 virus infection from December 2022 to Feb 2023 and followed up with them for 1 year. These children did not have severe respiratory, cardiovascular, or systemic symptoms. They were not given psychogenic drugs. We also searched Pubmed to identify previously reported acute onset psychiatric cases related to SARS-CoV-2 virus infection in children. We summarized these patients’ clinical symptoms, laboratory examination, treatment, and prognosis.

**Results:**

We reported 11 new cases of psychiatric disease directly related to SARS-CoV-2 virus infection and reviewed 12 previously reported cases among children and adolescents. They had various psychiatric symptoms within 3 weeks after the virus infection. Brain MRI and EEG recording did not reveal remarkable abnormalities. The cerebrospinal fluid analysis (CSF) could find increased protein, immunoglobulin, and IL-8 levels, disrupted blood–brain barrier, and positive oligoclonal band in a minority of the patients. Most of the patients had good outcomes.

**Conclusion:**

New-onset psychiatric symptoms directly related to SARS-CoV-2 virus infection are not rare phenomena among pediatric patients. CSF tests support the presence of central immune responses in some patients. Although these patients received different treatments, most of them had good prognoses.

## Introduction

There are many reports about mental disorders related to the COVID-19 pandemic since the spreading of the SARS-CoV-2 virus (1, 2). The pandemic-related stress caused an increased incidence of anxiety and depression (3). Psychotic symptoms such as hallucinations and delusions were highly prevalent during impairment of consciousness occurring in severe cases of SARS-CoV-2 virus infection (4). Iatrogenic factors, such as corticosteroids, sedatives, and anesthetic agents used during treatment for severe cases, can all have neuropsychiatric side effects. There were also some documents of new-onset psychoses supposed to be directly related to SARS-CoV-2 virus infection, most often from adult patients, rarely from children (1). Since December 2022, when China began implementing a relaxed anti-COVID-19 policy, our Pediatric neurology department has admitted several children/adolescents with psychiatric symptoms after being infected with the SARS-CoV-2 virus. We performed comprehensive examinations to identify possible etiologies that might contribute to psychiatric symptoms. However, we could not find any other etiologies that could explain the mental and behavioral abnormalities. We followed up with these patients and found that most patients’ symptoms disappeared, and only a small number of patients still had residual psychiatric symptoms. In this study, we report in detail the 11 pediatric cases with new onset psychiatric symptoms after SARS-CoV-2 virus infection and reviewed similar published cases in children and adolescents.

## Methods

From December 2022 to Feb 2023, young patients who presented to the pediatric neurology department of Qilu Hospital, Shandong University, with new onset psychiatric symptoms and evidence of a recent SARS-Cov-2 virus infection, were enrolled in this study. These patients did not experience hypoxia, altered consciousness, or corticosteroid administration before admission. Neurological examinations revealed normal orientation and attention which could exclude delirium status. Extensive examinations including blood count, liver and renal function, homocysteine level, blood ammonia and lactic acid level, thyroid function, rheumatic test, lymphocyte subset and immunoglobulin level, cerebrospinal fluid analysis, brain magnetic resonance imaging (MRI) and electroencephalogram (EEG) were performed to exclude other possible etiologies. We developed different treatment regimens for these young patients depending on the severity of the diseases. Immunosuppressive therapies were administered to most of our patients, considering that inflammatory reactions might be involved in the pathogenic pathway. We followed up with these patients for 1 year to collect possible residual symptoms.

We searched the Pubmed using the search terms ((“adolescent”[Mesh]) OR (“Child”[Mesh])) and ((“COVID-19”[Mesh]) OR (“SARS-CoV-2”[Mesh])) and (“mental disorders”[Mesh]) to identify relevant studies. By reading the titles and abstracts, we eliminated irrelevant articles that did not meet the inclusion criteria. Then, we evaluated the full-text articles to ensure they fulfilled the inclusion criteria and did not meet any of the exclusion criteria. We set inclusion criteria as follows: 1 children, adolescents or teenage patients, 2 patients with new onset psychiatric symptoms after or concurrent with documented SARS-CoV-2 virus infection. 3 articles with detailed descriptions of patient presentations. 4 articles written in English. Exclusion criteria include: 1 self-report and questionnaire study, 2 psychiatric symptoms that were not core symptoms, 3 psychiatric symptoms after the SARS-CoV-2 vaccine, 4 patients with multisystem inflammatory syndrome or severe respiratory illness, 5 patients in a decreased level of consciousness, 6 patients with positive autoimmune encephalitis antibody.

We extracted the following information from our patients and studies that met the review criteria: patient gender, age, past history, virus test method, the time interval between onset of respiratory symptoms and neuropsychiatric symptoms, psychiatric symptoms, blood test results, CSF analysis results, brain magnetic resonance imaging and other examinations, electroencephalography, clinical interventions and outcomes.

## Results

Case presentations (Table 1).

**Table 1:** Eleven pediatric cases of psychiatric symptoms after SARS-CoV-2 virus infection.

Case	Gender	Age	Past history	Virus detection	Time interval	Psychiatric symptoms	Blood test	CSF test	MRI	EEG	Intervention	follow-up
1	Male	13 Y	Mild intellectual disability	PCR	2 days	He could not take care of himself, but asked for help from his parents. Hyperlogia, insomnia, irritability.	Negative	Autoimmune encephalitis antibody and paraneoplastic antibody negative.	Not performed	Not performed	Olanzapine	Symptoms persist at 1 year’s follow-up.
2	Male	9 Y	Normal	PCR	Same day	Paroxysmal micropsia and teleopsia.	Negative	Autoimmune encephalitis antibody negative.	Normal	Normal	Methylprednisolone, IVIG	Symptoms completely resolved.
3	Female	2 Y	Normal	Antigen test	2 days	Auditory and visual hallucination. She saw clock swinging and heard something that did not exist. Nervous and fearful.	Negative	Autoimmune encephalitis antibody and oligoclonal band negative. CSF Cytokines normal.	Normal	Increased θ activity on the background	No treatment	Symptoms gradually resolved and disappeared.
4	Male	12 Y	Normal	Antigen test	6 days	Agitation, nonsense gibberish, involuntary limb movement, decreased and slow speech. He sometimes could not follow instructions.	Negative. Blood cytokines normal.	Increased albumin quotient and IgG. Autoimmune encephalitis antibody, paraneoplastic antibody, and oligoclonal band negative.	Normal	Atypical spikes, spike-slow waveson bilateral frontal lobe during sleep.	Methylprednisolone, IVIG, olanzapine	Symptoms mostly disappeared, only left with slow speech after treatment. After 1 year, he is completely normal.
5	Female	15 Y	Cerebral palsy	PCR	1 day	Nonsense gibberish, incommunicable, involuntary limb twisting, abnormal posture.	Negative. Blood cytokines normal.	Not performed.	Not performed	Not performed	Methylprednisolone, IVIG, olanzapine	Symptoms mostly resolved, almost returned to the status before illness.
6	Male	15 Y	Normal	PCR	12 days	Apathy, incommunicable, nervous with hand trembling, involuntary movement.	Negative. Blood cytokines normal.	Autoimmune encephalitis antibody, paraneoplastic antibody, and oligoclonal band negative.	Normal	Slowed background with dominant rhythm between 8 to 9 Hz	Methylprednisolone, IVIG	Symptoms completely disappeared.
7	Male	14 Y	Normal	PCR	7 days	Dreamful, poor sleep, agitation. Trembling hand, racing thoughts, nonsense gibberish, psychogenic nonepileptic seizures.	Negative Blood cytokines normal.	Autoimmune encephalitis antibody and oligoclonal band negative. Increased interleukin 8 in CSF.	Mega cisterna magna	Slowed background with dominant rhythm between 7 to 8 Hz	Methylprednisolone, IVIG, olanzapine	Symptoms mildly improved with better sleep. He is still excitable and sometimes has nonsense gibberish.
8	Female	10 Y	Normal	Antigen test & PCR	2 days	Drowsiness, fatigue, crying for no reason, agitation, unwilling to communicate.	ANA (±). Other blood test negative	Positive oligoclonal band in CSF. Autoimmune encephalitis antibody negative. CSF cytokines normal.	Normal	Slowed background	Methylprednisolone, IVIG	Symptoms mostly resolved after 5 days. She could communicate normally and had stable emotion. She still had memory impairment when she was discharged. She is completely normal at follow-up.
9	Female	15 Y	Normal	Antigen test	8 days	Nervousness, anxiety, insomnia, persecutory delusion, irascible, restless, unwilling to communicate, memory and computing ability impairment.	Anti-Ri antibody positive. Blood cytokines normal.	Autoimmune encephalitis antibody and paraneoplastic antibody negative. CSF cytokines normal.	Normal	Slowed background with dominant rhythm between 7-8 Hz	methylprednisolone, IVIG, valproate, olanzapine	Symptoms mostly resolved. She keeps reticent and still has memory and computing ability impairment.
10	Female	8 Y	Normal	Antigen test	2 weeks	Unwilling to communicate, decreased speech, insomnia, involuntary movement of face and limbs, laughing and shouting for no reason, childish behavior, abnormal gait and posture, episodic tachypnea.	Positive anti Ro-52, anti M2 antibody, and ANA after IVIG. Blood cytokines normal.	Autoimmune encephalitis antibody, paraneoplastic antibody, and oligoclonal band negative. CSF cytokine normal.	Normal	Normal	Methylprednisolone, IVIG	Symptoms partially improved. Reduced occurrence of mental and behavioral abnormalities. Improved communication. Occasional childish behavior and involuntary movement after 1 year.
11	Female	15 Y	Normal	PCR	2 days	Dizziness, fatigue, agitation, talking nonsense, visual hallucination, bizarre behavior.	Negative	Autoimmune encephalitis antibody and oligoclonal band negative. Increased interleukin 8 in CSF.	Normal	Normal	Methylprednisolone	Symptoms lasted shorter than 24 h and resolved before treatment.

### Case 1

A 13-year-old boy with mild intellectual disability lost the ability to take care of himself after 2 days of fever. He could not eat, dress, or go to the bathroom by himself. But he would like to ask his parents for help. He was dysphoric and talked too much in the daytime. Gradually, he developed insomnia. He was treated with olanzapine. However, he left the hospital against medical advice after 4 days of treatment. The boy’s symptoms persist after 1-year follow-up.

### Case 2

A 9-year-old boy experienced paroxysmal micropsia and teleopsia while having fever. Two days later, the bod did not have fever, but the visual symptoms persisted. The boy did not have headache attacks or seizures. Ophthalmic examinations were all normal. After treatment with intravenous immunoglobulin (IVIG, 2 g/kg) and 17 days of small-dose methylprednisolone, the boy’s visual symptoms disappeared.

### Case 3

A 2-year-old girl had fever for 2 days. After that, she said she could see the wall pendant swinging and hear strange sounds that she could not describe. Meanwhile, she felt scared and nervous. We did not give her medical intervention. The occurrence of visual and auditory hallucinations gradually decreased and eventually disappeared.

### Case 4

A 12-year-old boy had fever for 1 day, and then he had intermittent vomiting for one week three days after the fever. Six days after the fever, he was irritable with nonsense gibberish and involuntary limb movement. The boy gradually spoke little and slowly. The boy sometimes could not follow instructions. We gave him olanzapine, a small dose of methylprednisolone, and IVIG (2 g/kg). Most of his symptoms disappeared except slow speech after 15 days of treatment. After 1 year, he is completely normal.

### Case 5

A 15-year-old girl who had delayed psychomotor development due to cerebral palsy. She had fever for only 2 days. One day after fever, she became incommunicable and would like to talk nonsense gibberish. Involuntary limb movements and paroxysmal postural abnormalities could be easily observed. She received IVIG (2 g/kg) and a small dose of methylprednisolone for 14 days. The girl’s condition significantly improved and returned to the baseline level before virus infection at discharge and follow-up.

### Case 6

A 15-year-old boy had fever for 3 days. Twelve days after the fever, the child became apathetic with a dull expression. He did not want to communicate with other people. His hand would tremble when he was nervous, and he made involuntary tongue movements. We gave the patient high-dose methylprednisolone for 3 days (20 mg/kg/d) and gradually taper the dosage for a total of 14 days. At the same time, we treated the body with intravenous immunoglobulin (IVIG)(2 g/kg) over 4 days. After 14 days of treatment, the boy’s symptoms disappeared, and there was no relapse during long-term follow-up.

### Case 7

A 14-year-old boy had fever for only 1 day. One week later, he became dreamful and had poor sleep. Gradually, he developed symptoms of racing thoughts, nonsense gibberish, and agitation. He had one or more episodes of nonepileptic seizures every day: head nodding, eyes rolled up, hands trembling, and stiffness in the lower limbs. Response to olanzapine was poor. He received IVIG (2 g/kg) and 3 weeks of methylprednisolone pulse therapy (20 mg/kg/d for three consecutive days in 1 week). After 3 weeks of treatment, there was only improvement in sleep and no change in other symptoms. At 1 year’s follow-up, he is still excitable and sometimes talks nonsense gibberish.

### Case 8

A 10-year-old girl had fever for 1 day. She felt drowsiness and fatigue 2 days after fever. She was agitated and would cry for no reason. She would not like to talk with other people. She was given IVIG (2 g/kg) and 2 weeks of methylprednisolone pulse therapy (20 mg/kg/d for three consecutive days in 1 week). After treatment, the girl had stable emotion and normal communication with residual recent memory impairment. After 1 year, she is completely normal.

### Case 9

A 15-year-old girl became nervous, anxious, restless, and irascible at eightdays after having fever. She had insomnia and was unwilling to communicate with other people. She had the delusion of being hurt and blackmailed by other people. We found the girl’s memory and computing ability was impaired. Before admission, she was given valproate, olanzapine, and alprazolam. The insomnia improved, but other symptoms did not change. We asked her to discontinue alprazolam and still maintain on valproate and olanzapine. She received IVIG (2 g/kg) and 3 weeks of methylprednisolone pulse therapy (20 mg/kg/d for three consecutive days in 1 week).The girl improved with stable emotion, no delusions, and better sleep quality. However, memory and computing ability are only mildly improved and she is still reticent at 1 year’s follow-up.

### Case 10

An 8-year-old girl showed psychological and behavioral abnormalities 2 weeks after the SARS-CoV2 virus infection, including decreased speech, reluctance to communicate with others, irritability, insomnia, involuntary movement of face and limbs, laughing and shouting for no reason, childish behavior, abnormal gait and posture, episodic tachypnea.

She received a small dose of methylprednisolone for 12 days and left the hospital against medical advice. The girl had increased communication with her parents before discharge. The occurrence of involuntary movement and episodic tachypnea also decreased. After 1 year of follow-up, the girl still has childish behavior and occasional involuntary movement.

### Case 11

A 15-year-old girl had fever and sore throat for 2 days. Immediately after the temperature dropped to normal, she felt dizziness and fatigue. She was irritable and talking nonsense the whole night. She reported seeing his father who was not there. She also had the bizarre behavior of checking the windows over and over again, bending over the floor to count non-existing beads. Her symptoms only lasted for 12 h and resolved spontaneously. We still empirically administered 14 days of small-dose methylprednisolone. After discharge, She has been living a normal life.

### Summary of our cases

Among the 11 patients, five were male and six were female. The children’s ages ranged from 2 to 15 years old. They have psychiatric symptoms within 2 weeks after infection of SARS-CoV2 virus. All children underwent extensive blood tests to rule out metabolic factors, rheumatic factors, and immunodeficiency syndromes. Six children were tested for blood cytokine levels, all of which were within the normal ranges (S1). All patients underwent rheumatic antibody test panel and eight of them were all negative. One patient was weakly positive for ANA. Another patient was positive for both anti-Ro-52 antibody and ANA. The last patient was positive for anti-Ro-52, anti-M2, and ANA after IVIG infusion. Lumbar puncture was performed in 10 children. Eight children showed no sign of pleocytosis or increased protein levels. One child had increased IgG level and albumin quotient in CSF. Another child was positive for the oligoclonal band in CSF. All 10 children tested negative for autoimmune encephalitis antibodies in blood and CSF. Six children were tested for paraneoplastic antibodies, and one of them had positive anti-Ri antibody in the blood. Cerebrospinal fluid cytokines were tested in six patients, and only two patients had elevated interleukin 8 (IL-8) levels (S1). Brain MRI was unremarkable in the nine patients who had completed the examination. The ophthalmic examination was performed on the child with visual symptoms to rule out ophthalmologic disorders. Nine children underwent EEG monitoring, of which four had slow background rhythm, one had increased *θ* rhythm activity, one had spike wave, and three had completely normal EEG. Five children were treated with corticosteroids or corticosteroids combined with IVIG. Four children were given oral antipsychotic drugs along with corticosteroids and IVIG. One child was given only oral antipsychotic drug. One child did not receive any treatment. After long-term follow-up, one child had persistent symptoms, one child had slightly improved symptoms, one child had partially improved symptoms, and eight children had significant improvement or complete remission.

### Summary of reviewed articles

The detailed clinical information are documented in Table 2 (5–13). There were a total of 9 articles which met the search criteria, including 12 cases. All of them were children older than 10 years old or adolescents. Six patients were male, three patients were female, and the genders of the remaining three patients were unavailable. The detailed time intervals between SARS-CoV-2 virus infection and onset of psychiatric symptoms were provided for nine patients. Psychiatric symptoms can occur concurrently with the viral infection or as long as 3 weeks after the virus infection. Blood test results were mentioned in eight patients. One patient had positive ANA. Two patients with obsessive-compulsive disorder symptoms had positive anti-basal ganglia antibodies. One patient had both positive anti-GAD antibody and anti-Ro antibody after IVIG infusion. There were detailed descriptions of cerebrospinal spinal fluid examination in nine patients, six of which had normal test results. Two patients had increased protein levels, positive anti-SARS-COV2-spike protein IgG, and anti-neural antibodies in cerebrospinal fluid. One patient had positive oligoclonal bands in CSF. MRI scannings were normal or nonspecific for all of the 11 patients who performed MRI examinations. Ten patients underwent EEG examination, and six of them were normal. One patient had diffuse *β* activity, and three patients had increased slow-wave activity. Four children were given antipsychotic drugs. Four children were treated with antipsychotic drugs, corticosteroids, and IVIG. One child was treated with antipsychotic drugs and corticosteroids. Two patients received psychological intervention. One child did not receive any treatment. After treatment, two children had persistent symptoms, one child had only slight improvement, two children had partial improvement, and seven children had significant improvement or complete remission of symptoms.

**Table 2:** Reviewed child and adolescent cases of psychiatric symptoms after SARS-CoV-2 virus infection.

Study	Gender	Age	Past history	Virus detection	Time interval	Psychiatric Symptoms	Blood test	CSF test	MRI and others	EEG	Intervention	Follow-up
Ref (5)	Female	16 Y	Mild learning needs	PCR	3 days	Anxious, insomnia, paranoia, ritualistic behaviors, visual and auditory hallucinations, mutism, no voluntary activity. Fed via nasogastric tube. Fecal and urinary incontinence.	Anti-GAD and anti-Ro antibody positive post IVIG.	Autoimmune encephalitis panel, oligoclonal band, and infectious screen negative.	Nonspecific T2 flair hyperintensities in the centrum semiovale bilaterally.	δ slowing, more prominent in the right hemisphere posteriorly.	Olanzapine, haloperidol, benzodiazepine, IVIG of 2 g/kg, methylprednisolone 1 g over 3 days.	Significantly improved after 6 months
Ref (6)	male	12 Y	No movement disorder or neuropsychiatric disturbances	PCR	2 weeks after testing positive	OCD (severe drive to wash his hands, emotional lability, facial motor tics)	Anti-basal ganglia antibody positive.	NA	Normal	Normal	Psychological intervention	Symptoms persist
Ref (6)	male	13 Y	NA	PCR	13 days	OCD disorder (using only a tablespoon during his meals and arranging the tip of his shoes in parallel before going to sleep), tics, hyperactivity, aggressiveness, irritability, inattentiveness, inappetence.	Anti-basal ganglia antibody positive.	NA	Normal	Normal	Psychological intervention	Symptoms persist, more aggressive and more irritable
Ref (7)	NA	Teenaged	Marijuana use, unspecified anxiety and depression	PCR	NA	Subacute social withdrawal, insomnia, erratic behavior, paranoia, delusion.	NA	Elevated protein levels and elevated IgG index. IgG against SARS-CoV-2 spike protein. Positive antineural antibodies. Autoimmune encephalopathy panel negative.	Nonspecific T2 white matter hyper intensities in the frontal lobes.	Normal	Valproate, lorazepam and gabapentin, IVIG 2 g/kg, methylprednisolone 1 g per day for 3 days and a prednisone taper.	The patient had more organized thoughts, decreased paranoia, and improved insight. More than 1 month after symptom onset, the patient’s delusions and hyperreflexia had resolved; however, the patient’s lability persisted, and he was discharged taking valproate.
Ref (7)	NA	Teenaged	Unspecified anxiety and motor tics, endorsed foggy brain	Positive SARS-CoV-2-IgG serology	Concurrently	Word finding difficulties, impaired concentration and difficulty completing homework, insomnia, and mood lability characterized by sobbing and elation. The patient experienced internal preoccupation, aggression, and suicidal ideation.	NA	Elevated protein levels. IgG against SARS-CoV-2 spike protein. Positive antineural antibodies.	Normal	Normal	Quetiapine, Lithium, methylprednisolone 1 g per day for 5 days. IVIG 2 g/kg over 3 days.	Although improved from initial presentation, the patient required academic accommodations and continued to endorse forgetfulness and attention difficulties.
Ref (7)	NA	Teenaged	No known psychiatric history	PCR	NA	Repetitive behavior, anorexia, insomnia, tachypneic, ideomotor apraxia, abulia, disorganized behavior, agitation.	NA	Three unique oligoclonal bands in CSF.	Normal	Diffuse β activity	Olanzapine, lorazepam	Discharged normally without medications.
Ref (8)	Male	17 Y	No psychiatric history	PCR	3 weeks	Bizarre behavior, paranoid delusions, auditory hallucination, agitation, aggression, emotional lability, insomnia.	NA	NA	NA	NA	Aripiprazole	Significant improvement and no relapse
Ref (9)	Male	10 Y	Normal psychomotor development	PCR	NA	Hallucination, restlessness, aggression, self-harming thoughts.	Negative	Autoimmune encephalitis antibodies negative.	Lateral ventricular asymmetry on MRI. Ophthalmologic examination normal.	Episodic θ activity mixing above the centro-parietal and posterior areas.	Clonazepam, alprazolamclarithromycin, methylprednisolone	Symptoms disappeared, back to normal life.
Ref (10)	Female	15 Y	No past psychiatric history and no significant family history	PCR	2.5 weeks	Insomnia, acting erratically, hallucination, delusion, seizure-like activity, paranoia, internally occupied.	Negative	Autoimmune antibody negative. Meningitis and encephalitis panel negative.	Normal	Unremarkable	Olanzapine	Symptoms all resolved
Ref (11)	Male	16 Y	History of allergy	Antigen test	Concurrently	Attempt to interact with items that were not there, forgetful, hypersexuality, transient memory loss.	Negative	Negative	Normal	Normal	No treatment	Recalled the episode and expressed both anger and sadness.
Ref (12)	Male	16 Y	Normal development	Antigen test	9 days	Anxious, agitated, swear at parents, unusual gestures, hallucination, delusion, fast tangential speech, suicidal ideation.	Negative	Negative	Normal	Not performed	Haloperidol and lorazepam, olanzapine.	Exhibit no psychiatric signs, returned to his premorbid state.
Ref (13)	Female	11 Y	Learning, attention and fine motor difficulties and mild anxiety.	PCR	3 weeks	Visual and auditory hallucinations, anxiety, extreme fear, aggression, confusion with disorganized behavior.	Positive ANA 1:320 nuclear dot pattern. Positive antibodies to SARS-CoV-2 spike glycoprotein	Autoimmune encephalopathy panel negative.	Normal	Intermittent short epochs of bilateral left>right temporal slowing	Haloperidol. 5-day course of prednisone 40 mg/d, 2 g/kg IVIG twice with intermittent time of 4 weeks.	Mild improvement.

## Discussion

Acute psychosis has long been associated with respiratory viral pandemics, such as influenza (H1N1), SARS, and MERS (14–18). Neuropsychiatric symptoms can occur in the context of acute viral infection or after varied periods of time. Since the outset of SARS-CoV-2 virus pandemic, there have been increases in newly-onset mental and psychiatric disorders which might be caused by a combination of factors. We categorize these different factors into the following three groups. The first group of patients develop depression, anxiety, sleep disorders, and PTSD due to isolation, stress, and panic during the pandemic event (19–21). The second group of patients had severe cardiopulmonary disease or systemic diseases induced by SARS-CoV-2 virus infection and developed neuropsychiatric symptoms. Both delirium/encephalopathy status and psychogenic treatment can contribute to increased psychiatric symptoms (22). The third group of patients is similar to our cases, which indicate that acute-onset psychiatric symptoms are directly related to SARS-CoV-2 virus infection (23). SARS-CoV-2 virus caused only mild respiratory symptoms or no symptoms, but patients developed a variety of psychiatric symptoms.

Most of the reports in the third group are adult cases (1). We found 12 pediatric cases and summarized their clinical data. Detailed information shows that these children developed psychiatric symptoms within 3 weeks of virus infection. Three children (3/8, 37%) had peripheral immune responses on blood antibody tests before immunoglobulin transfusion. Three children (3/9, 33%) had elevated protein level, anti-neural antibodies, or positive oligoclonal bands in cerebrospinal fluid, suggesting central inflammatory responses. Magnetic resonance examinations were normal or showed nonspecific abnormalities. EEG revealed increased fast activity in one child (1/10, 10%) and increased slow wave activity in three children (3/10, 30%). Although these children received different treatments, most of them had significant improvement or complete resolution (7/12, 58%).

Our child patients shared many similarities with previously reported cases. Our patients developed psychiatric symptoms within 2 weeks of virus infection. A small number (3/11, 27%) of our children had positive antibodies in the blood before immunoglobulin infusion, suggesting a peripheral immune response. In a small portion of patients (2/10, 20%), positive findings in CSF suggest intracranial immune response and disruption of the blood–brain barrier. Magnetic resonance imaging did not have revealing findings. EEG showed epileptic wave in one child (1/9, 11%) and slow background rhythm or slow wave activities in five children (5/9, 55%). We chose different therapies for these children according to the severity of the symptoms. The psychiatric symptoms significantly improved or entirely resolved in most of these children (8/11, 72%).

Several potential mechanisms have been proposed for the neuropsychiatric sequelae of SARS-CoV-2 virus infection: 1. SARS-CoV-2 virus can enter the central nervous system directly through the olfactory (24) or hematogenous route (25, 26), although the virus RNA could rarely be detected in the cerebrospinal fluid (27). 2. SARS-Cov-2 virus-infected microglia are potential mediators of neurological problems by producing pro-inflammatory cytokines, such as IL-1β, IL-6, and TNF-*α* (28). 3. Activation of peripheral cytokines may precipitate inflammation of the central nervous system and disruption of the blood–brain barrier (29). 4. Inflammatory cytokines could inhibit enzymes responsible for the synthesis of basic compounds of cerebral neurotransmission, inducing psychiatric symptoms (30). 5. Viral infection could induce abnormal immune responses that might attack self-antigens (31), which could be further supported by the virus infection-associated anti-NMDAR encephalitis cases (32).

There were evidences that pro-inflammatory cytokines are elevated in patients with SARS-CoV-2 virus infection and can reflect disease severity, including IL-6, IL-8, IL-10, IL-2R, and TNF *α* (33–35). Blood cytokine tests were performed in six of our patients, and all were within the normal range. No cardinal systemic symptoms at the time of tests could explain the absence of inflammatory cytokine storm in the blood. Cytokine levels were also measured in CSF in six children, and one-third (2/6, 33.3%) had increased IL-8 levels, which indicates IL-8 in the central nervous system may be involved in the mechanisms of SARS-CoV-2 virus-induced psychiatric symptoms. In the brain, microglia and astrocytes can release IL-8 in response to oxidative stress or psychosocial stressors (36). Increased IL-8 levels in CSF have been reported in brain infection and head injury (37, 38). Blood IL-8 levels are also elevated in bipolar disorder, schizophrenia, obstructive sleep apnea and autism spectrum disorder (36). How IL-8 affected brain function and caused a wide variety of psychiatric symptoms in patients infected with SARS-CoV-2 virus is unclear. Moreover, only a minority of our patients had increased IL-8 levels in CSF, indicating pathogenic mechanisms other than IL-8. We also detected elevated IgG level, albumin quotient, and positive oligoclonal band in the CSF in another two children, which supports the disrupted blood–brain barrier and central autoimmune reaction theory. Increased protein levels, positive anti-neural antibodies and positive oligoclonal bands in CSF from previously reported three-child cases also support the central autoimmune mechanisms.

In summary, we encountered 11 children with various psychiatric symptoms directly related to SARS-CoV-2 virus infection during the first 3 months of the COVID-19 pandemic from late 2022 to early 2023, which suggests that new onset psychiatric disease after SARS-CoV-2 virus infection is not a rare phenomenon among children. Blood tests denied the existence of peripheral cytokine storm, and only a minority of patients had elevated IL-8 in the CSF. Evidence of central immune responses could be found in the cerebrospinal fluid from some of our children and previously reported cases. Although there is no consensus on the treatment for these children, most of them had good prognoses.

## Data Availability

The original contributions presented in the study are included in the article/Supplementary material, further inquiries can be directed to the corresponding author.
