# Supplementary material for: Acute onset psychiatric diseases after SARS-CoV-2 virus infection among pediatric patients
Source: Front Neurol. 2024 Oct 9;15:1445903. doi: 10.3389/fneur.2024.1445903 (PMC11496280; doi:10.3389/fneur.2024.1445903)

Blood cytokines levels in six of our children are listed in Table 1, and they are all within the normal range. CSF cytokine levels in six of our children are listed in Table 2. Most cytokines were below detectable levels or just above detectable level(IL-6), except that IL-8 was remarkably higher than detectable level. However, we do not know the normal reference value for IL-8 in the cerebrospinal fluid. So, we retrospectively collected IL-8 levels in CSF from 96 children who were admitted for headache and underwent lumbar puncture to rule out intracranial inflammation and intracranial hypertension. These 96 values were proved to be normally distributed by the Kolmogorov-Smirnov test(P=0.09, Fig 1). Then we calculated the mean value(‾X) and standard deviation(S.D.)(Fig 1). We set the upper limit of normal IL-8 level in CSF as ‾X+1.645×S.D., which equals to 82.64pg/ml. IL-8 levels in CSF from case 7 and case 11 were higher than the upper limit of normal value.

Table 1 Blood cytokines levels in six of our children

| Cytokines  (normal range) | Case 4 | Case 5 | Case 6 | Case 7 | Case 9 | Case 10 |
| --- | --- | --- | --- | --- | --- | --- |
| IL-1β (≤12.4pg/ml) | <2.5 | <2.5 | <2.5 | <2.5 | <2.5 | <2.5 |
| IL-2 (≤5.71pg/ml) | <2.5 | <2.5 | <2.5 | <2.5 | <2.5 | <2.5 |
| IL-4 (≤3.00pg/ml) | <2.5 | <2.5 | <2.5 | <2.5 | <2.5 | <2.5 |
| IL-5 (≤3.10pg/ml) | <2.5 | <2.5 | <2.5 | <2.5 | <2.5 | <2.5 |
| IL-6 (≤5.30pg/ml) | <2.5 | <2.5 | 2.63 | <2.5 | <2.5 | <2.5 |
| IL-8 (≤53.09pg/ml) | 3.5 | 7.99 | <2.5 | <2.5 | <2.5 | <2.5 |
| IL-10 (≤4.91pg/ml) | <2.5 | <2.5 | <2.5 | <2.5 | <2.5 | <2.5 |
| IL-12p70 (≤3.40pg/ml) | <2.5 | <2.5 | <2.5 | <2.5 | <2.5 | <2.5 |
| IL-17 (≤20.60pg/ml) | <10.0 | <10.0 | <10.0 | <10.0 | <10.0 | <10.0 |
| IFN 𝛼 (≤8.50pg/ml) | <2.5 | <2.5 | <2.5 | <2.5 | <2.5 | 3.02 |
| IFN 𝛾 (≤7.42pg/ml) | <2.5 | <2.5 | <2.5 | <2.5 | <2.5 | <2.5 |
| TNF 𝛼 (≤4.60pg/ml) | <2.5 | <2.5 | <2.5 | <2.5 | <2.5 | <2.5 |

Table 2 CSF cytokines levels in six of our children

| Cytokines  (pg/ml) | Case 3 | Case 7 | Case 8 | Case 9 | Case 10 | Case 11 |
| --- | --- | --- | --- | --- | --- | --- |
| IL-1β | <2.5 | <2.5 | <2.5 | <2.5 | <2.5 | <2.5 |
| IL-2 | <2.5 | <2.5 | <2.5 | <2.5 | <2.5 | <2.5 |
| IL-4 | <2.5 | <2.5 | <2.5 | <2.5 | <2.5 | <2.5 |
| IL-5 | <2.5 | <2.5 | <2.5 | <2.5 | <2.5 | <2.5 |
| IL-6 | 3.06 | <2.5 | 3.24 | <2.5 | <2.5 | 3.15 |
| IL-8 | 14.68 | 322.10 | 61.53 | 72.13 | 40.77 | 186.26 |
| IL-10 | <2.5 | <2.5 | <2.5 | <2.5 | <2.5 | <2.5 |
| IL-12p70 | <2.5 | <2.5 | <2.5 | <2.5 | <2.5 | <2.5 |
| IL-17 | <10.0 | <10.0 | <10.0 | <10.0 | <10.0 | <10.0 |
| IFN 𝛼 | <2.5 | <2.5 | <2.5 | <2.5 | <2.5 | <2.5 |
| IFN 𝛾 | <2.5 | <2.5 | <2.5 | <2.5 | <2.5 | <2.5 |
| TNF 𝛼 | <2.5 | <2.5 | <2.5 | <2.5 | <2.5 | <2.5 |


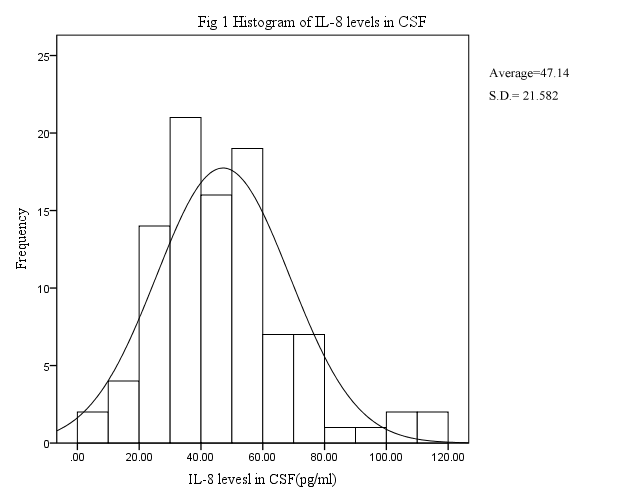

Supplement: Supplementary file 1 [file Data_Sheet_1.docx]
